# BPA and PXR Activation: Human Receptor Is Affected, Mouse Receptor Is Not

**DOI:** 10.1289/ehp.120-a122a

**Published:** 2012-03-01

**Authors:** Julia R. Barrett

**Affiliations:** Julia R. Barrett, MS, ELS, a Madison, WI–based science writer and editor, has written for *EHP* since 1996. She is a member of the National Association of Science Writers and the Board of Editors in the Life Sciences.

The high-production-volume chemical bisphenol A (BPA) is a key component in polycarbonate plastics and is used in a wide variety of consumer products. The U.S. general population is widely exposed to BPA, as demonstrated by the chemical’s presence in more than 90% of biological samples tested. BPA has been associated with cardiovascular disease and diabetes in human population studies, but if causality were to be proven, it is doubtful it would arise from BPA’s known weak interaction with estrogen receptors. Researchers previously determined that BPA targets another nuclear receptor, the pregnane X receptor (PXR), and have now determined that the chemical and several related compounds (“analogues”) can induce metabolically important genes via PXR activation [*EHP* 120(3):399–405; Sui et al.].

PXR is activated by numerous endogenous and environmental chemicals. Earlier research established that PXR activation in mice and rats induces genes involved in lipid homeostasis, atherosclerosis, and carcinogenesis. Although PXR is not affected by BPA in rodents, the chemical activates the human receptor and induces target gene expression. This connection suggested a possible mechanism to explain BPA-related cardiovascular disease findings in human populations.

In the current study, transfection assays using mouse- and human-derived receptors showed that BPA induces strong, dose-dependent activation of human PXR but not mouse PXR. Computational docking and modeling studies focusing on the structure of human PXR identified key amino acids within the ligand-binding domain that permit BPA to interact with the receptor. These studies also highlighted how species-related differences in this domain can affect how different species respond to specific chemicals.

The modeled predictions were confirmed *in vitro* using site-directed mutagenesis assays that focused on the amino acids critical for BPA docking within PXR. Additionally, several BPA structural analogues were found to activate the PXR. Experiments to determine interactions between BPA and specific analogues showed synergism when BPA was paired with 2-(4'-hydroxyphenyl)-2-phenylpropane (HPP); that is, the effect of the mixture exceeded the sum of the individual chemicals. In a final set of experiments, the ability of BPA and analogues HPP and bisphenol B to separately trigger expression of PXR target genes was tested in a human intestinal cell line. All 3 compounds were found to trigger expression of the metabolic genes *CYP3A4*, *UGT1A1*, and *MDR1*.

Although this study did not address questions of risk assessment, its findings suggest that BPA may adversely affect human health via PXR and thus provide a foundation for further exploration of the observed association between BPA and cardiovascular disease. Additionally, given the likelihood that real-life exposures involve chemical mixtures, the demonstrated synergism between BPA and HPP supports the need to include mixtures in *in vivo* experiments.

